# Hexaaqua­nickel(II) tetra­aqua­bis­(μ-pyridine-2,6-dicarboxyl­ato)bis­(pyridine-2,6-dicarboxyl­ato)trinickelate(II) octa­hydrate

**DOI:** 10.1107/S1600536810028977

**Published:** 2010-07-24

**Authors:** Javad Safaei-Ghomi, Elham Motieiyan, Faranak Manteghi, Mohammad Ghadermazi, Hossein Aghabozorg

**Affiliations:** a Department of Chemistry, Islamic Azad University, Qom Branch, Qom, Iran; b Department of Chemistry, Faculty of Science, Payame Noor University (PNU), Qom, Iran; c Department of Chemistry, Iran University of Science and Technology, Tehran, Iran; d Department of Chemistry, Faculty of Science, University of Kurdistan, Sanandaj, Iran; e Department of Chemistry, Islamic Azad University, North Tehran Branch, Tehran, Iran

## Abstract

The title compound, [Ni(H_2_O)_6_][Ni_3_(C_7_H_3_NO_4_)_4_(H_2_O)_4_]·8H_2_O, was obtained by the reaction of nickel(II) nitrate hexa­hydrate with pyridine-2,6-dicarb­oxy­lic acid (pydcH_2_) and 1,10-phenanothroline (phen) in an aqueous solution. The latter ligand is not involved in formation of the title complex. There are three different Ni^II^ atoms in the asymmetric unit, two of which are located on inversion centers, and thus the [Ni(H_2_O)_6_]^2+^ cation and the trinuclear {[Ni(pydc)_2_]_2_-μ-Ni(H_2_O)_4_}^2−^ anion are centrosymmetric. All Ni^II^ atoms exhibit an octa­hedral coordination geometry. Various inter­actions, including numerous O—H⋯O and C—H⋯O hydrogen bonds and C—O⋯π stacking of the pyridine and carboxyl­ate groups [3.570 (1), 3.758 (1) and 3.609 (1) Å], are observed in the crystal structure.

## Related literature

For metal complexes formed by pyridine­dicarb­oxy­lic acids, see: Aghabozorg *et al.* (2008 ▶); Çolak *et al.* (2008 ▶); Moghimi *et al.* (2005 ▶).
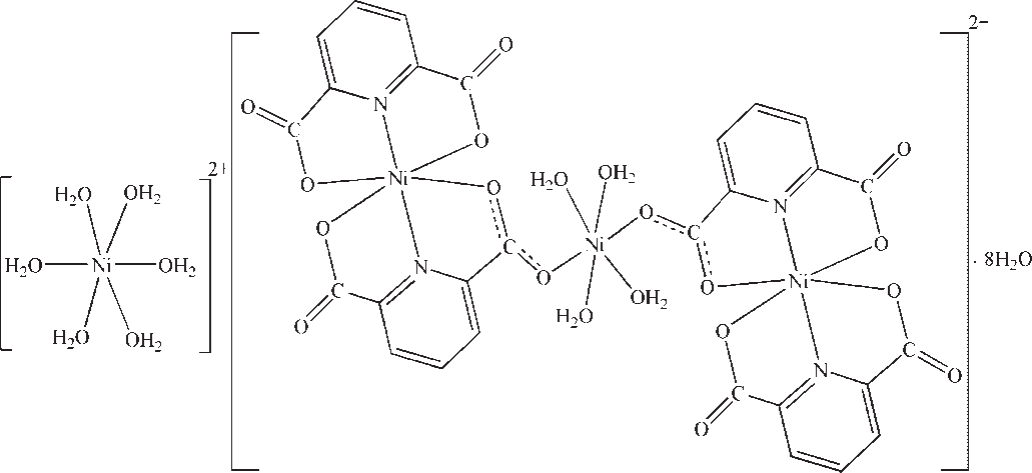


## Experimental

### 

#### Crystal data

[Ni(H_2_O)_6_][Ni_3_(C_7_H_3_NO_4_)_4_(H_2_O)_4_]·8H_2_O
*M* *_r_* = 1219.54
Monoclinic, 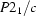

*a* = 20.4561 (5) Å
*b* = 12.7587 (3) Å
*c* = 8.8582 (2) Å
β = 96.942 (1)°
*V* = 2294.98 (9) Å^3^
*Z* = 2
Mo *K*α radiation
μ = 1.73 mm^−1^
*T* = 100 K
0.35 × 0.13 × 0.07 mm

#### Data collection

Bruker APEXII CCD area-detector diffractometer
Absorption correction: multi-scan (*SADABS*; Bruker, 2005 ▶) *T*_min_ = 0.760, *T*_max_ = 0.888
27378 measured reflections
6068 independent reflections
4899 reflections with *I* > 2σ(*I*)
*R*_int_ = 0.062

#### Refinement

*R*[*F* ^2^ > 2σ(*F* ^2^)] = 0.027
*wR*(*F* ^2^) = 0.064
*S* = 1.00
6068 reflections
319 parameters
H-atom parameters constrained
Δρ_max_ = 0.83 e Å^−3^
Δρ_min_ = −0.62 e Å^−3^

### 

Data collection: *APEX2* (Bruker, 2005 ▶); cell refinement: *SAINT* (Bruker, 2005 ▶); data reduction: *SAINT*; program(s) used to solve structure: *SHELXTL* (Sheldrick, 2008 ▶); program(s) used to refine structure: *SHELXTL*; molecular graphics: *SHELXTL*; software used to prepare material for publication: *SHELXTL*.

## Supplementary Material

Crystal structure: contains datablocks I, global. DOI: 10.1107/S1600536810028977/gk2279sup1.cif

Structure factors: contains datablocks I. DOI: 10.1107/S1600536810028977/gk2279Isup2.hkl

Additional supplementary materials:  crystallographic information; 3D view; checkCIF report

## Figures and Tables

**Table 1:** Hydrogen-bond geometry (Å, °)

*D*—H⋯*A*	*D*—H	H⋯*A*	*D*⋯*A*	*D*—H⋯*A*
O1*W*—H1⋯O5	0.85	2.12	2.852 (2)	143
O1*W*—H2⋯O6*W*^i^	0.85	1.92	2.756 (2)	169
O2*W*—H3⋯O4^i^	0.85	1.95	2.787 (2)	166
O2*W*—H4⋯O6*W*^ii^	0.85	1.87	2.724 (2)	178
O3*W*—H5⋯O7	0.85	2.05	2.891 (2)	173
O3*W*—H6⋯O9*W*^i^	0.85	1.94	2.789 (2)	180
O4*W*—H7⋯O2^iii^	0.85	1.97	2.767 (2)	155
O4*W*—H8⋯O9*W*	0.85	1.94	2.782 (2)	175
O5*W*—H9⋯O8	0.85	1.84	2.690 (2)	175
O5*W*—H10⋯O7*W*^iv^	0.85	1.88	2.722 (2)	170
O6*W*—H11⋯O4	0.85	1.85	2.670 (2)	162
O6*W*—H12⋯O8*W*^v^	0.85	1.93	2.777 (2)	172
O7*W*—H13⋯O8	0.85	1.86	2.707 (2)	173
O7*W*—H14⋯O1^iv^	0.85	1.94	2.760 (2)	163
O8*W*—H15⋯O1*W*^v^	0.85	2.43	3.106 (2)	137
O8*W*—H16⋯O3	0.85	1.95	2.787 (2)	166
O9*W*—H17⋯O7*W*^vi^	0.85	1.88	2.705 (2)	164
O9*W*—H18⋯O2^vii^	0.85	1.98	2.779 (2)	156
C2—H2*A*⋯O3*W*^i^	0.95	2.37	3.266 (2)	157
C9—H9*A*⋯O8*W*^viii^	0.95	2.38	3.183 (2)	143

Symmetry codes: (i) 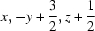; (ii) 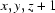; (iii) 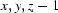; (iv) 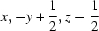; (v) 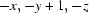; (vi) 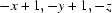; (vii) 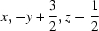; (viii) 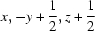.

**Fig. 1.**
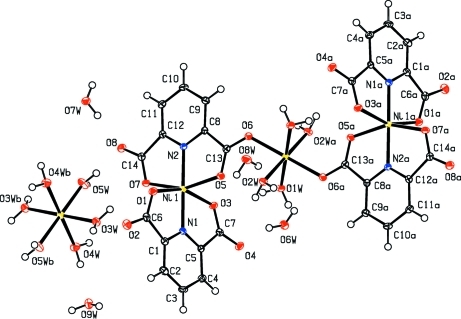
View of the title compound with displacement ellipsoids at the 50 % probability level. Symmetry codes to generate equivalent atoms: #a -x, -y+1, -z+1; #b -x+1,-y+1,-z.

**Fig. 2.**
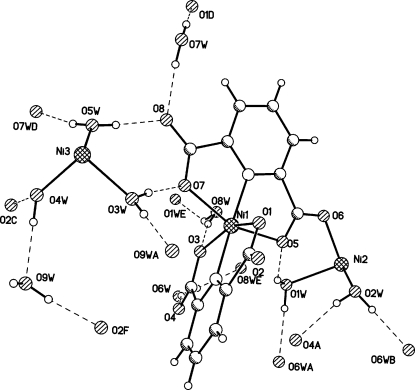
Hydrogen bonding pattern. Hydrogen bonds are shown with dashed lines.Symmetry transformations used to generate equivalent atoms: #A x, -y + 3/2; z + 1/2; #B x, y, z + 1; #C x, y, z - 1; #D x, -y + 1/2, z - 1/2; #E -x, -y + 1, -z; #F x, -y + 3/2, z - 1/2.

**Fig. 3.**
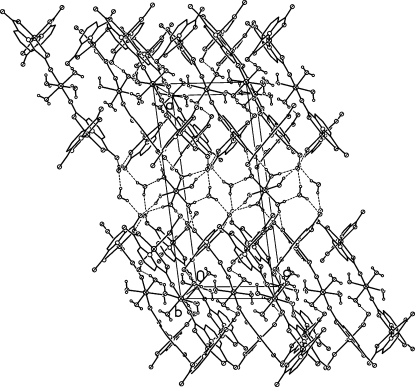
Crystal packing fragment along the b crystal axis. Hydrogen bonds are shown with dashed lines. Only H atoms that take part in hydrogen bonding are depicted for clarity.

## supplementary crystallographic information

### Comment

Pyridinedicarboxylic acid and 1,10-phenanthroline are well known ligands in coordination chemistry (Çolak *et al.*, 2008). In our group, many compounds are synthesized by proton transfer between the two compounds (phenH)_2_(pydc) (Moghimi *et al.*, 2005). Also, many metallic compounds have been reported (Aghabozorg *et al.*, 2008).

The considerable case in the title compound is that despite of the presence of phen in the preparation solution, only pydc is involved in the complex formation. In the crystal structure, the Ni atom is in three types. In the anionic part, Ni1 is coordinated by two (pydc)^2– ^groups, and Ni2 is coordinated by four water molecules and two uncoordinated O atoms of two (pydc)^2– ^groups linked to Ni1. As shown in Fig.1, this causes that Ni2 makes a bridge between Ni1 and Ni1A. In the cationic part, Ni3 is simply coordinated by six water molecules.

As given in Table 1 and Figs. 2–3, there are nomerous hydrogen bonds of the type O—H···O between water molecules and O atoms of (pydc)^2–^, and C—H···O between C atoms of pyridine rings and water molecules. Also, C—O···π stackings present in the crystal structure, are as follows: C6—O2···*Cg*1(N1/C1—C5), 3.570 (1) Å, C14—O8···*Cg*2 (N2/C8—C12), 3.758 (1) Å, and C7—O4···*Cg*1(N1/C1—C5), 3.609 (1) Å.

### Experimental

To an aqueous solution of Ni(NO_3_)_2_.6H_2_O (143 mg, 0.5 mmol), an aqueous solution of pydcH_2_ (167 mg, 1 mmol) and phen (198 mg, 1 mmol) in 1:2:2 molar ratio was added. The final volume was 40 ml. After less than 1 h stirring and heating, the obtained clear solution was left for 2 weeks. Then emerald green crystals were settled in the solution which were suitable for X-ray crystallography.

### Refinement

The H atoms of the water molecules were found in difference Fourier maps and the O-H bond lengths were constrained to 0.85 Å. The H atoms from C-H groups were placed in calculated positions. All H atoms were refined in riding model approximation with *U*_iso_(H) = 1.2U*eq*(C) or 1.5U_eq_(O).

### Crystal data

**Table:**

[Ni(H_2_O)_6_][Ni_3_(C_7_H_3_NO_4_)_4_(H_2_O)_4_]·8H_2_O	*F*(000) = 1256
*M**_r_* = 1219.54	*D*_x_ = 1.765 Mg m^−^^3^
Monoclinic, *P*2_1_/*c*	Mo *K*α radiation, λ = 0.71073 Å
Hall symbol: -P 2ybc	Cell parameters from 8450 reflections
*a* = 20.4561 (5) Å	θ = 2.6–33.9°
*b* = 12.7587 (3) Å	µ = 1.73 mm^−^^1^
*c* = 8.8582 (2) Å	*T* = 100 K
β = 96.942 (1)°	Prism, green
*V* = 2294.98 (9) Å^3^	0.35 × 0.13 × 0.07 mm
*Z* = 2	

### Data collection

**Table:**

Bruker APEXII CCD area-detector diffractometer	6068 independent reflections
Radiation source: fine-focus sealed tube	4899 reflections with *I* > 2σ(*I*)
graphite	*R*_int_ = 0.062
ω scans	θ_max_ = 29.0°, θ_min_ = 1.9°
Absorption correction: multi-scan (*SADABS*; Bruker, 2005)	*h* = −27→27
*T*_min_ = 0.760, *T*_max_ = 0.888	*k* = −17→17
27378 measured reflections	*l* = −12→12

### Refinement

**Table:**

Refinement on *F*^2^	Primary atom site location: structure-invariant direct methods
Least-squares matrix: full	Secondary atom site location: difference Fourier map
*R*[*F*^2^ > 2σ(*F*^2^)] = 0.027	Hydrogen site location: mixed
*wR*(*F*^2^) = 0.064	H-atom parameters constrained
*S* = 1.00	*w* = 1/[σ^2^(*F*_o_^2^) + (0.025*P*)^2^] where *P* = (*F*_o_^2^ + 2*F*_c_^2^)/3
6068 reflections	(Δ/σ)_max_ = 0.001
319 parameters	Δρ_max_ = 0.83 e Å^−^^3^
0 restraints	Δρ_min_ = −0.62 e Å^−^^3^

### Special details

**Table:**

Geometry. All e.s.d.'s (except the e.s.d. in the dihedral angle between two l.s. planes) are estimated using the full covariance matrix. The cell e.s.d.'s are taken into account individually in the estimation of e.s.d.'s in distances, angles and torsion angles; correlations between e.s.d.'s in cell parameters are only used when they are defined by crystal symmetry. An approximate (isotropic) treatment of cell e.s.d.'s is used for estimating e.s.d.'s involving l.s. planes.
Refinement. Refinement of *F*^2^ against ALL reflections. The weighted *R*-factor *wR* and goodness of fit *S* are based on *F*^2^, conventional *R*-factors *R* are based on *F*, with *F* set to zero for negative *F*^2^. The threshold expression of *F*^2^ > σ(*F*^2^) is used only for calculating *R*-factors(gt) *etc*. and is not relevant to the choice of reflections for refinement. *R*-factors based on *F*^2^ are statistically about twice as large as those based on *F*, and *R*- factors based on ALL data will be even larger.

### Fractional atomic coordinates and isotropic or equivalent isotropic displacement parameters (Å^2^)

**Table:**

	*x*	*y*	*z*	*U*_iso_*/*U*_eq_	
Ni1	0.231007 (11)	0.520201 (16)	0.27476 (3)	0.00945 (6)	
Ni2	0.0000	0.5000	0.5000	0.00895 (7)	
Ni3	0.5000	0.5000	0.0000	0.01050 (7)	
O1	0.30490 (6)	0.51969 (9)	0.46564 (14)	0.0142 (3)	
O2	0.38195 (6)	0.62577 (10)	0.58535 (14)	0.0168 (3)	
O3	0.16507 (6)	0.58836 (9)	0.09653 (14)	0.0136 (3)	
O4	0.12370 (6)	0.74544 (9)	0.02196 (14)	0.0152 (3)	
O5	0.14709 (6)	0.51140 (9)	0.39541 (14)	0.0131 (3)	
O6	0.06993 (6)	0.39623 (9)	0.44857 (14)	0.0124 (3)	
O7	0.30351 (6)	0.46255 (9)	0.14135 (14)	0.0130 (3)	
O8	0.33719 (6)	0.31334 (9)	0.04162 (14)	0.0159 (3)	
N1	0.25177 (7)	0.66974 (11)	0.29750 (16)	0.0100 (3)	
N2	0.20957 (7)	0.37003 (10)	0.26000 (16)	0.0095 (3)	
C1	0.29991 (8)	0.69995 (13)	0.40384 (19)	0.0111 (3)	
C2	0.31588 (9)	0.80472 (14)	0.4262 (2)	0.0145 (4)	
H2A	0.3507	0.8258	0.5008	0.017*	
C3	0.27913 (9)	0.87836 (13)	0.3356 (2)	0.0147 (4)	
H3A	0.2887	0.9509	0.3485	0.018*	
C4	0.22850 (9)	0.84595 (13)	0.2262 (2)	0.0130 (4)	
H4A	0.2030	0.8954	0.1643	0.016*	
C5	0.21645 (8)	0.73898 (13)	0.21023 (19)	0.0101 (3)	
C6	0.33232 (8)	0.60828 (14)	0.4934 (2)	0.0124 (3)	
C7	0.16388 (8)	0.68773 (13)	0.09888 (19)	0.0111 (3)	
C8	0.15876 (8)	0.33432 (13)	0.32572 (19)	0.0102 (3)	
C9	0.14264 (9)	0.22888 (13)	0.3229 (2)	0.0132 (4)	
H9A	0.1062	0.2038	0.3694	0.016*	
C10	0.18182 (9)	0.16074 (13)	0.2493 (2)	0.0141 (4)	
H10A	0.1725	0.0878	0.2467	0.017*	
C11	0.23432 (8)	0.19915 (13)	0.1799 (2)	0.0130 (4)	
H11A	0.2609	0.1533	0.1288	0.016*	
C12	0.24700 (8)	0.30604 (13)	0.18706 (19)	0.0102 (3)	
C13	0.12210 (8)	0.42038 (13)	0.39702 (19)	0.0108 (3)	
C14	0.30067 (8)	0.36433 (13)	0.11794 (19)	0.0112 (3)	
O1W	0.01802 (6)	0.59383 (9)	0.31474 (14)	0.0138 (3)	
H1	0.0598	0.5956	0.3192	0.021*	
H2	0.0099	0.6586	0.3247	0.021*	
O2W	0.06619 (6)	0.58178 (9)	0.64628 (14)	0.0132 (3)	
H3	0.0894	0.6282	0.6090	0.020*	
H4	0.0493	0.6161	0.7140	0.020*	
O3W	0.43024 (6)	0.56054 (9)	0.13011 (14)	0.0146 (3)	
H5	0.3947	0.5269	0.1346	0.022*	
H6	0.4439	0.5949	0.2099	0.022*	
O4W	0.47679 (6)	0.61160 (9)	−0.16482 (14)	0.0161 (3)	
H7	0.4418	0.6029	−0.2257	0.024*	
H8	0.4738	0.6773	−0.1511	0.024*	
O5W	0.43241 (6)	0.39907 (10)	−0.10299 (14)	0.0191 (3)	
H9	0.4006	0.3750	−0.0599	0.029*	
H10	0.4215	0.3998	−0.1987	0.029*	
O6W	0.00996 (6)	0.69298 (10)	−0.14073 (14)	0.0161 (3)	
H11	0.0463	0.6959	−0.0832	0.024*	
H12	−0.0169	0.6531	−0.1024	0.024*	
O7W	0.39596 (6)	0.12448 (9)	0.09304 (14)	0.0155 (3)	
H13	0.3764	0.1819	0.0690	0.023*	
H14	0.3737	0.0707	0.0622	0.023*	
O8W	0.06730 (6)	0.44404 (10)	−0.00502 (15)	0.0195 (3)	
H15	0.0422	0.4701	−0.0791	0.029*	
H16	0.0967	0.4913	0.0103	0.029*	
O9W	0.47481 (6)	0.82603 (10)	−0.10844 (14)	0.0179 (3)	
H17	0.5157	0.8380	−0.0864	0.027*	
H18	0.4564	0.8473	−0.0330	0.027*	

### Atomic displacement parameters (Å^2^)

**Table:**

	*U*^11^	*U*^22^	*U*^33^	*U*^12^	*U*^13^	*U*^23^
Ni1	0.01011 (11)	0.00744 (10)	0.01080 (11)	−0.00024 (8)	0.00129 (8)	−0.00014 (8)
Ni2	0.00992 (15)	0.00820 (15)	0.00908 (15)	−0.00061 (11)	0.00256 (12)	−0.00069 (11)
Ni3	0.01054 (15)	0.01220 (16)	0.00858 (15)	−0.00051 (12)	0.00045 (12)	0.00130 (12)
O1	0.0152 (6)	0.0122 (6)	0.0146 (6)	0.0005 (5)	−0.0003 (5)	0.0015 (5)
O2	0.0147 (6)	0.0218 (7)	0.0128 (6)	−0.0014 (5)	−0.0025 (5)	0.0018 (5)
O3	0.0146 (6)	0.0090 (6)	0.0166 (7)	0.0002 (5)	−0.0012 (5)	−0.0017 (5)
O4	0.0155 (6)	0.0123 (6)	0.0164 (7)	0.0028 (5)	−0.0035 (5)	0.0008 (5)
O5	0.0130 (6)	0.0095 (6)	0.0175 (6)	−0.0009 (5)	0.0051 (5)	−0.0022 (5)
O6	0.0121 (6)	0.0111 (6)	0.0145 (6)	−0.0003 (5)	0.0035 (5)	−0.0005 (5)
O7	0.0152 (6)	0.0094 (6)	0.0148 (6)	−0.0010 (5)	0.0038 (5)	0.0011 (5)
O8	0.0177 (6)	0.0133 (6)	0.0185 (7)	0.0018 (5)	0.0098 (6)	−0.0004 (5)
N1	0.0103 (7)	0.0098 (7)	0.0103 (7)	−0.0004 (5)	0.0022 (6)	−0.0008 (5)
N2	0.0103 (7)	0.0087 (7)	0.0092 (7)	−0.0001 (5)	−0.0004 (6)	0.0000 (5)
C1	0.0112 (8)	0.0130 (8)	0.0096 (8)	−0.0011 (6)	0.0023 (7)	−0.0004 (6)
C2	0.0151 (9)	0.0157 (9)	0.0125 (9)	−0.0038 (7)	0.0013 (7)	−0.0024 (7)
C3	0.0184 (9)	0.0084 (8)	0.0177 (9)	−0.0027 (7)	0.0041 (8)	−0.0029 (7)
C4	0.0153 (9)	0.0113 (8)	0.0132 (9)	0.0013 (7)	0.0047 (7)	0.0013 (7)
C5	0.0096 (8)	0.0107 (8)	0.0104 (8)	0.0003 (6)	0.0031 (7)	0.0003 (6)
C6	0.0116 (8)	0.0162 (9)	0.0101 (8)	0.0005 (7)	0.0045 (7)	0.0012 (7)
C7	0.0117 (8)	0.0120 (8)	0.0102 (8)	−0.0004 (7)	0.0042 (7)	−0.0008 (6)
C8	0.0096 (8)	0.0114 (8)	0.0096 (8)	0.0005 (6)	0.0014 (7)	0.0001 (6)
C9	0.0139 (9)	0.0116 (8)	0.0145 (9)	−0.0014 (7)	0.0037 (7)	0.0000 (7)
C10	0.0182 (9)	0.0081 (8)	0.0162 (9)	−0.0006 (7)	0.0026 (7)	−0.0013 (7)
C11	0.0143 (9)	0.0109 (8)	0.0141 (9)	0.0026 (7)	0.0021 (7)	−0.0013 (7)
C12	0.0098 (8)	0.0122 (8)	0.0084 (8)	−0.0004 (6)	0.0005 (6)	0.0004 (6)
C13	0.0115 (8)	0.0116 (8)	0.0087 (8)	0.0011 (6)	−0.0007 (7)	−0.0007 (6)
C14	0.0113 (8)	0.0134 (8)	0.0087 (8)	0.0005 (7)	0.0008 (7)	0.0020 (6)
O1W	0.0148 (6)	0.0107 (6)	0.0165 (6)	−0.0003 (5)	0.0043 (5)	0.0015 (5)
O2W	0.0148 (6)	0.0121 (6)	0.0136 (6)	−0.0033 (5)	0.0050 (5)	−0.0021 (5)
O3W	0.0131 (6)	0.0161 (6)	0.0144 (6)	−0.0019 (5)	0.0011 (5)	−0.0013 (5)
O4W	0.0197 (7)	0.0149 (6)	0.0124 (6)	0.0005 (5)	−0.0029 (5)	0.0033 (5)
O5W	0.0198 (7)	0.0267 (7)	0.0110 (6)	−0.0101 (6)	0.0033 (5)	−0.0017 (5)
O6W	0.0154 (6)	0.0170 (6)	0.0155 (7)	−0.0023 (5)	0.0002 (5)	−0.0024 (5)
O7W	0.0160 (6)	0.0114 (6)	0.0183 (7)	−0.0004 (5)	−0.0008 (5)	−0.0009 (5)
O8W	0.0191 (7)	0.0159 (6)	0.0228 (7)	−0.0042 (5)	−0.0005 (6)	−0.0017 (5)
O9W	0.0145 (6)	0.0222 (7)	0.0173 (7)	0.0009 (5)	0.0028 (5)	0.0011 (5)

### Geometric parameters (Å, °)

**Table:**

Ni1—N1	1.9598 (14)	C4—C5	1.391 (2)
Ni1—N2	1.9663 (14)	C4—H4A	0.9500
Ni1—O1	2.1275 (12)	C5—C7	1.517 (2)
Ni1—O5	2.1319 (12)	C8—C9	1.385 (2)
Ni1—O3	2.1338 (12)	C8—C13	1.510 (2)
Ni1—O7	2.1357 (13)	C9—C10	1.396 (2)
Ni2—O6	2.0407 (12)	C9—H9A	0.9500
Ni2—O2W	2.0438 (12)	C10—C11	1.390 (2)
Ni2—O1W	2.0996 (12)	C10—H10A	0.9500
Ni3—O5W	2.0242 (12)	C11—C12	1.388 (2)
Ni3—O4W	2.0535 (12)	C11—H11A	0.9500
Ni3—O3W	2.0879 (12)	C12—C14	1.515 (2)
O1—C6	1.273 (2)	O1W—H1	0.8500
O2—C6	1.243 (2)	O1W—H2	0.8499
O3—C7	1.268 (2)	O2W—H3	0.8500
O4—C7	1.244 (2)	O2W—H4	0.8499
O5—C13	1.270 (2)	O3W—H5	0.8498
O6—C13	1.249 (2)	O3W—H6	0.8500
O7—C14	1.270 (2)	O4W—H7	0.8500
O8—C14	1.249 (2)	O4W—H8	0.8500
N1—C5	1.329 (2)	O5W—H9	0.8499
N1—C1	1.335 (2)	O5W—H10	0.8500
N2—C8	1.332 (2)	O6W—H11	0.8500
N2—C12	1.338 (2)	O6W—H12	0.8500
C1—C2	1.385 (2)	O7W—H13	0.8499
C1—C6	1.520 (2)	O7W—H14	0.8501
C2—C3	1.395 (2)	O8W—H15	0.8500
C2—H2A	0.9500	O8W—H16	0.8499
C3—C4	1.393 (2)	O9W—H17	0.8501
C3—H3A	0.9500	O9W—H18	0.8500
			
N1—Ni1—N2	177.85 (6)	C1—C2—C3	117.79 (16)
N1—Ni1—O1	78.28 (5)	C1—C2—H2A	121.1
N2—Ni1—O1	100.44 (5)	C3—C2—H2A	121.1
N1—Ni1—O5	100.22 (5)	C4—C3—C2	120.27 (16)
N2—Ni1—O5	78.19 (5)	C4—C3—H3A	119.9
O1—Ni1—O5	98.00 (5)	C2—C3—H3A	119.9
N1—Ni1—O3	77.82 (5)	C5—C4—C3	118.01 (16)
N2—Ni1—O3	103.41 (5)	C5—C4—H4A	121.0
O1—Ni1—O3	156.09 (5)	C3—C4—H4A	121.0
O5—Ni1—O3	85.25 (5)	N1—C5—C4	121.07 (15)
N1—Ni1—O7	103.64 (5)	N1—C5—C7	112.62 (14)
N2—Ni1—O7	77.99 (5)	C4—C5—C7	126.30 (15)
O1—Ni1—O7	87.97 (5)	O2—C6—O1	126.32 (16)
O5—Ni1—O7	156.12 (5)	O2—C6—C1	118.31 (15)
O3—Ni1—O7	98.63 (5)	O1—C6—C1	115.37 (15)
O6—Ni2—O6^i^	180.0	O4—C7—O3	126.56 (16)
O6—Ni2—O2W	92.53 (5)	O4—C7—C5	118.08 (15)
O6^i^—Ni2—O2W	87.47 (5)	O3—C7—C5	115.35 (14)
O6—Ni2—O2W^i^	87.47 (5)	N2—C8—C9	121.43 (16)
O6^i^—Ni2—O2W^i^	92.53 (5)	N2—C8—C13	112.79 (14)
O2W—Ni2—O2W^i^	180.00 (5)	C9—C8—C13	125.77 (16)
O6—Ni2—O1W^i^	89.92 (5)	C8—C9—C10	117.68 (16)
O6^i^—Ni2—O1W^i^	90.08 (5)	C8—C9—H9A	121.2
O2W—Ni2—O1W^i^	87.76 (5)	C10—C9—H9A	121.2
O2W^i^—Ni2—O1W^i^	92.24 (5)	C11—C10—C9	120.33 (16)
O6—Ni2—O1W	90.08 (5)	C11—C10—H10A	119.8
O6^i^—Ni2—O1W	89.92 (5)	C9—C10—H10A	119.8
O2W—Ni2—O1W	92.24 (5)	C12—C11—C10	118.45 (16)
O2W^i^—Ni2—O1W	87.76 (5)	C12—C11—H11A	120.8
O1W^i^—Ni2—O1W	180.0	C10—C11—H11A	120.8
O5W—Ni3—O5W^ii^	180.00 (6)	N2—C12—C11	120.45 (16)
O5W—Ni3—O4W^ii^	88.05 (5)	N2—C12—C14	112.37 (14)
O5W^ii^—Ni3—O4W^ii^	91.95 (5)	C11—C12—C14	127.18 (15)
O5W—Ni3—O4W	91.95 (5)	O6—C13—O5	126.35 (16)
O5W^ii^—Ni3—O4W	88.05 (5)	O6—C13—C8	117.58 (15)
O4W^ii^—Ni3—O4W	180.00 (5)	O5—C13—C8	116.05 (15)
O5W—Ni3—O3W	90.51 (5)	O8—C14—O7	125.70 (16)
O5W^ii^—Ni3—O3W	89.49 (5)	O8—C14—C12	118.23 (15)
O4W^ii^—Ni3—O3W	88.78 (5)	O7—C14—C12	116.06 (15)
O4W—Ni3—O3W	91.22 (5)	Ni2—O1W—H1	104.4
O5W—Ni3—O3W^ii^	89.49 (5)	Ni2—O1W—H2	114.8
O5W^ii^—Ni3—O3W^ii^	90.51 (5)	H1—O1W—H2	100.1
O4W^ii^—Ni3—O3W^ii^	91.22 (5)	Ni2—O2W—H3	117.9
O4W—Ni3—O3W^ii^	88.78 (5)	Ni2—O2W—H4	114.6
O3W—Ni3—O3W^ii^	180.0	H3—O2W—H4	102.0
C6—O1—Ni1	113.90 (11)	Ni3—O3W—H5	119.0
C7—O3—Ni1	114.02 (10)	Ni3—O3W—H6	118.1
C13—O5—Ni1	113.80 (11)	H5—O3W—H6	114.8
C13—O6—Ni2	125.07 (11)	Ni3—O4W—H7	117.7
C14—O7—Ni1	114.14 (11)	Ni3—O4W—H8	126.6
C5—N1—C1	121.44 (15)	H7—O4W—H8	98.6
C5—N1—Ni1	119.45 (11)	Ni3—O5W—H9	123.0
C1—N1—Ni1	119.08 (11)	Ni3—O5W—H10	121.7
C8—N2—C12	121.66 (14)	H9—O5W—H10	109.2
C8—N2—Ni1	118.94 (11)	H11—O6W—H12	110.4
C12—N2—Ni1	119.38 (11)	H13—O7W—H14	113.4
N1—C1—C2	121.41 (16)	H15—O8W—H16	101.3
N1—C1—C6	112.60 (14)	H17—O9W—H18	106.2
C2—C1—C6	125.99 (16)		
			
N1—Ni1—O1—C6	−7.59 (12)	C2—C3—C4—C5	−0.4 (3)
N2—Ni1—O1—C6	174.17 (12)	C1—N1—C5—C4	0.2 (3)
O5—Ni1—O1—C6	−106.44 (12)	Ni1—N1—C5—C4	178.12 (13)
O3—Ni1—O1—C6	−10.1 (2)	C1—N1—C5—C7	−179.19 (14)
O7—Ni1—O1—C6	96.78 (12)	Ni1—N1—C5—C7	−1.30 (19)
N1—Ni1—O3—C7	−7.77 (12)	C3—C4—C5—N1	0.5 (3)
N2—Ni1—O3—C7	170.42 (12)	C3—C4—C5—C7	179.86 (16)
O1—Ni1—O3—C7	−5.3 (2)	Ni1—O1—C6—O2	−170.33 (14)
O5—Ni1—O3—C7	93.77 (12)	Ni1—O1—C6—C1	9.82 (19)
O7—Ni1—O3—C7	−109.96 (12)	N1—C1—C6—O2	173.09 (15)
N1—Ni1—O5—C13	179.05 (11)	C2—C1—C6—O2	−7.7 (3)
N2—Ni1—O5—C13	−2.43 (11)	N1—C1—C6—O1	−7.0 (2)
O1—Ni1—O5—C13	−101.49 (11)	C2—C1—C6—O1	172.18 (17)
O3—Ni1—O5—C13	102.36 (11)	Ni1—O3—C7—O4	−169.34 (14)
O7—Ni1—O5—C13	1.74 (19)	Ni1—O3—C7—C5	9.26 (18)
O2W—Ni2—O6—C13	−56.66 (13)	N1—C5—C7—O4	173.03 (15)
O2W^i^—Ni2—O6—C13	123.34 (13)	C4—C5—C7—O4	−6.4 (3)
O1W^i^—Ni2—O6—C13	−144.42 (13)	N1—C5—C7—O3	−5.7 (2)
O1W—Ni2—O6—C13	35.58 (13)	C4—C5—C7—O3	174.92 (17)
N1—Ni1—O7—C14	177.23 (11)	C12—N2—C8—C9	0.6 (2)
N2—Ni1—O7—C14	−1.32 (11)	Ni1—N2—C8—C9	−178.02 (13)
O1—Ni1—O7—C14	99.80 (12)	C12—N2—C8—C13	−178.15 (14)
O5—Ni1—O7—C14	−5.49 (19)	Ni1—N2—C8—C13	3.20 (18)
O3—Ni1—O7—C14	−103.30 (11)	N2—C8—C9—C10	0.4 (3)
O1—Ni1—N1—C5	−174.37 (14)	C13—C8—C9—C10	178.99 (16)
O5—Ni1—N1—C5	−78.23 (13)	C8—C9—C10—C11	−1.0 (3)
O3—Ni1—N1—C5	4.59 (12)	C9—C10—C11—C12	0.6 (3)
O7—Ni1—N1—C5	100.65 (13)	C8—N2—C12—C11	−1.0 (2)
O1—Ni1—N1—C1	3.57 (12)	Ni1—N2—C12—C11	177.63 (12)
O5—Ni1—N1—C1	99.72 (13)	C8—N2—C12—C14	178.50 (14)
O3—Ni1—N1—C1	−177.47 (13)	Ni1—N2—C12—C14	−2.85 (18)
O7—Ni1—N1—C1	−81.40 (13)	C10—C11—C12—N2	0.4 (3)
O1—Ni1—N2—C8	95.39 (12)	C10—C11—C12—C14	−179.07 (16)
O5—Ni1—N2—C8	−0.69 (12)	Ni2—O6—C13—O5	14.8 (2)
O3—Ni1—N2—C8	−82.83 (12)	Ni2—O6—C13—C8	−163.58 (11)
O7—Ni1—N2—C8	−178.97 (13)	Ni1—O5—C13—O6	−173.65 (13)
O1—Ni1—N2—C12	−83.29 (13)	Ni1—O5—C13—C8	4.74 (18)
O5—Ni1—N2—C12	−179.38 (13)	N2—C8—C13—O6	173.22 (14)
O3—Ni1—N2—C12	98.49 (13)	C9—C8—C13—O6	−5.5 (3)
O7—Ni1—N2—C12	2.34 (12)	N2—C8—C13—O5	−5.3 (2)
C5—N1—C1—C2	−1.1 (3)	C9—C8—C13—O5	175.96 (16)
Ni1—N1—C1—C2	−179.00 (13)	Ni1—O7—C14—O8	178.91 (14)
C5—N1—C1—C6	178.18 (15)	Ni1—O7—C14—C12	0.24 (18)
Ni1—N1—C1—C6	0.27 (19)	N2—C12—C14—O8	−177.18 (15)
N1—C1—C2—C3	1.2 (3)	C11—C12—C14—O8	2.3 (3)
C6—C1—C2—C3	−178.01 (17)	N2—C12—C14—O7	1.6 (2)
C1—C2—C3—C4	−0.4 (3)	C11—C12—C14—O7	−178.94 (16)

Symmetry codes: (i) −*x*, −*y*+1, −*z*+1; (ii) −*x*+1, −*y*+1, −*z*.

### Hydrogen-bond geometry (Å, °)

**Table:**

*D*—H···*A*	*D*—H	H···*A*	*D*···*A*	*D*—H···*A*
O1W—H1···O5	0.85	2.12	2.852 (2)	143
O1W—H2···O6W^iii^	0.85	1.92	2.756 (2)	169
O2W—H3···O4^iii^	0.85	1.95	2.787 (2)	166
O2W—H4···O6W^iv^	0.85	1.87	2.724 (2)	178
O3W—H5···O7	0.85	2.05	2.891 (2)	173
O3W—H6···O9W^iii^	0.85	1.94	2.789 (2)	180
O4W—H7···O2^v^	0.85	1.97	2.767 (2)	155
O4W—H8···O9W	0.85	1.94	2.782 (2)	175
O5W—H9···O8	0.85	1.84	2.690 (2)	175
O5W—H10···O7W^vi^	0.85	1.88	2.722 (2)	170
O6W—H11···O4	0.85	1.85	2.670 (2)	162
O6W—H12···O8W^vii^	0.85	1.93	2.777 (2)	172
O7W—H13···O8	0.85	1.86	2.707 (2)	173
O7W—H14···O1^vi^	0.85	1.94	2.760 (2)	163
O8W—H15···O1W^vii^	0.85	2.43	3.106 (2)	137
O8W—H16···O3	0.85	1.95	2.787 (2)	166
O9W—H17···O7W^ii^	0.85	1.88	2.705 (2)	164
O9W—H18···O2^viii^	0.85	1.98	2.779 (2)	156
C2—H2A···O3W^iii^	0.95	2.37	3.266 (2)	157
C9—H9A···O8W^ix^	0.95	2.38	3.183 (2)	143

Symmetry codes:  (iii) *x*, −*y*+3/2, *z*+1/2; (iv) *x*, *y*, *z*+1; (v) *x*, *y*, *z*−1; (vi) *x*, −*y*+1/2, *z*−1/2; (vii) −*x*, −*y*+1, −*z*; (ii) −*x*+1, −*y*+1, −*z*; (viii) *x*, −*y*+3/2, *z*−1/2; (ix) *x*, −*y*+1/2, *z*+1/2.
